# Severity of Peripheral Infection Differentially Affects Brain Functions in Mice via Microglia-Dependent and -Independent Mechanisms

**DOI:** 10.3390/ijms242417597

**Published:** 2023-12-18

**Authors:** Yen-Phung Le, Kozo Saito, Bijay Parajuli, Kent Sakai, Yuto Kubota, Miho Miyakawa, Youichi Shinozaki, Eiji Shigetomi, Schuichi Koizumi

**Affiliations:** 1 Department of Neuropharmacology, Interdisciplinary Graduate School of Medicine, University of Yamanashi, Chuo 409-3898, Japan; g20ddm25@yamanashi.ac.jp (Y.-P.L.); ksaitoko@yamanashi.ac.jp (K.S.); parajuli@yamanashi.ac.jp (B.P.); ksakai@yamanashi.ac.jp (K.S.); g22dim02@yamanashi.ac.jp (Y.K.); mihomi@yamanashi.ac.jp (M.M.); shinozaki-yi@igakuken.or.jp (Y.S.); eshigetomi@yamanashi.ac.jp (E.S.); 2 GLIA Center, University of Yamanashi, Chuo 409-3898, Japan

**Keywords:** microglia, macrophage, ischemic tolerance, middle cerebral artery occlusion (MCAO), lipopolysaccharide (LPS), RNA sequencing

## Abstract

Peripheral infection induces inflammation in peripheral tissues and the brain, impacting brain function. Glial cells are key players in this process. However, the effects of peripheral infection on glial activation and brain function remain unknown. Here, we showed that varying degrees of peripheral infection had different effects on the regulation of brain functions by microglia-dependent and -independent mechanisms. Acute mild infection (one-day LPS challenge: 1LPS) exacerbated middle cerebral artery occlusion (MCAO) injury, and severe infection (four-day LPS challenge: 4LPS) for one week suppressed it. MCAO injury was assessed by triphenyltetrazolium chloride staining. We observed early activation of microglia in the 1LPS and 4LPS groups. Depleting microglia with a colony-stimulating factor-1 receptor (CSF1R) antagonist had no effect on 1LPS-induced brain injury exacerbation but abolished 4LPS-induced protection, indicating microglial independence and dependence, respectively. Microglia-independent exacerbation caused by 1LPS involved peripheral immune cells including macrophages. RNA sequencing analysis of 4LPS-treated microglia revealed increased factors related to anti-inflammatory and neuronal tissue repair, suggesting their association with the protective effect. In conclusion, varying degrees of peripheral inflammation had contradictory effects (exacerbation vs. protection) on MCAO, which may be attributed to microglial dependence. Our findings highlight the significant impact of peripheral infection on brain function, particularly in relation to glial cells.

## 1. Introduction

Infection and inflammation in peripheral tissues have a significant impact on central functions. Although viruses and bacteria can infiltrate the brain directly, in most cases various molecules such as danger-associated molecular patterns (DAMPs) and cytokines released from peripheral immune cells reach the brain and affect brain functions [1,2]. In addition to neurons, the brain contains vascular system cells as well as glial cells, which have an important role in communication with peripheral immune cells [3,4]. Glial cells in the brain include astrocytes, oligodendrocytes, and especially microglia, which have an important role in information exchange and are considered interface cells with peripheral organs and cells. For example, microglia in the spinal cord dorsal horn sense peripheral nerve injury and transmit it to the brain, causing neuropathic pain [5]. Microglia can also sense systemic peripheral inflammatory conditions via the blood-brain barrier, and communicate them to the brain [6].

Microglia respond immediately to various types of peripheral infection and inflammation such as the intraperitoneal administration of lipopolysaccharide (LPS), which mimics bacterial infection in the periphery, by changing their phenotype to a strongly Iba1-positive activated form [2]. Microglia are the only innate immune cells in the brain and are very sensitive to environmental changes inside and outside the brain. In addition to their function as immune cells, microglia are involved in synaptogenesis [7], synaptic pruning [8], environmental maintenance by debris removal [9], protective effects in neurodegenerative diseases [10], as well as playing a very important role in the regulation of core brain functions. Therefore, if the microglia phenotype changes upon receiving peripheral inflammatory information, the ability of the brain to transmit information is greatly affected. However, little is known about the regulation of brain functions via microglia during peripheral infection/inflammation. In particular, it is unclear how peripheral inflammation triggered by LPS affects microglia and alters pathological conditions such as ischemic damage.

In this study, we used a model that mimics peripheral infection using LPS to evaluate how microglia perceive a peripheral infection and to investigate the subsequent effects on brain damage induced by middle cerebral artery occlusion (MCAO). We also investigated the effects of different degrees of peripheral inflammation on brain function by varying the dose of LPS. Here, we showed that mild LPS stimulation (one-day LPS challenge) exacerbated subsequent MCAO-induced brain damage and that this was microglia-independent. We also showed that severe LPS stimulation (four-day LPS challenge), unlike mild LPS stimulation, inhibited MCAO-induced brain infarction via a microglia-dependent mechanism, and demonstrated the molecular mechanism involved. Thus, we demonstrate that peripheral infection and inflammation regulate MCAO damage in opposite directions, depending on the degree of infection and inflammation, and that the presence or absence of microglial involvement has a major role in these processes.

## 2. Results

### 2.1. Opposite Effects of Different Doses of LPS on MCAO-Evoked Brain Damage

**Figure 1:**
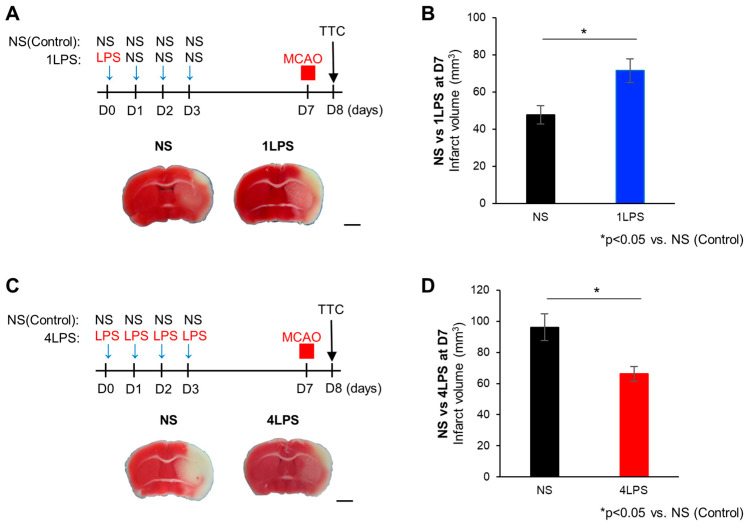
LPS-induced mild or severe inflammation influences MCAO infarct lesion size. (**A**) Top: Schematic diagram showing the experimental protocol. Bottom: Representative images of triphenyltetrazolium chloride (TTC) staining in the normal saline (NS) group (left) and 1LPS group (right). Scale bar, 2 mm. (**B**) Quantification of infarct volumes (NS group (*n* = 5 mice) vs. 1LPS mice (*n* = 6 mice), * *p* < 0.05, unpaired *t*-test). (**C**) Top: Schematic diagram showing the experimental protocol. Bottom: Representative images of TTC staining in the NS group (left) and 4LPS group (right). Scale bar, 2 mm. (**D**) Quantification of infarct volumes (NS group (*n* = 7 mice) vs. 4LPS mice (*n* = 8 mice), * *p* < 0.05, unpaired *t*-test). All data are expressed as the mean ± standard error of the mean (SEM).

To examine the effect of different degrees of peripheral inflammation on brain ischemic insults, we administered LPS before the induction of experimental brain injury by MCAO. We injected C57BL6/J mice with a single dose of 500 µg/kg LPS or normal saline intraperitoneally (i.p.), followed by normal saline (NS) injection for three consecutive days (hereafter 1LPS mice and NS mice, respectively; see Section 4 Materials and Methods). Seven days after the first injection (D7), MCAO was performed for 30 min, followed by triphenyltetrazolium chloride (TTC) staining the next day to assess the infarct volume (Figure 1A). We found larger infarct volumes in the MCAO ipsilateral hemisphere of 1LPS-treated mice than in mice receiving NS injection, suggesting that LPS treatment exacerbated the cerebral infarction (Figure 1B). To investigate whether severe inflammation affected the ischemic lesions, we treated mice with NS or LPS (500 µg/kg) once per day for four consecutive days, and then performed MCAO for 40 min (Figure 1C). Interestingly, mice with a four-day LPS challenge (4LPS mice) had reduced infarct volumes compared with four-day NS-treated control mice (NS group). In the NS group, infarct lesions were present in the cortex and striatum, but in the 4LPS group, the lesions were dramatically decreased, especially in the striatum (Figure 1D; control NS group for 4LPS is substantially different from that for the 1LPS group because the MCAO duration time of 1LPS and 4LPS were not identical; see Section 4 Materials and Methods). These data suggest that severe inflammation (4LPS) may have an inhibitory effect on ischemic damage, whereas mild peripheral inflammation (1LPS) worsens it. The 1LPS-evoked MCAO exacerbation and 4LPS-evoked MCAO protection were transient responses as they were observed at D7 but had disappeared by D14. Thus, the degree of peripheral inflammation might determine the amelioration or augmentation of brain infarctions.

### 2.2. LPS Administration Induces the Transient Activation of Microglia and Sustained Activation of Astrocytes

**Figure 2:**
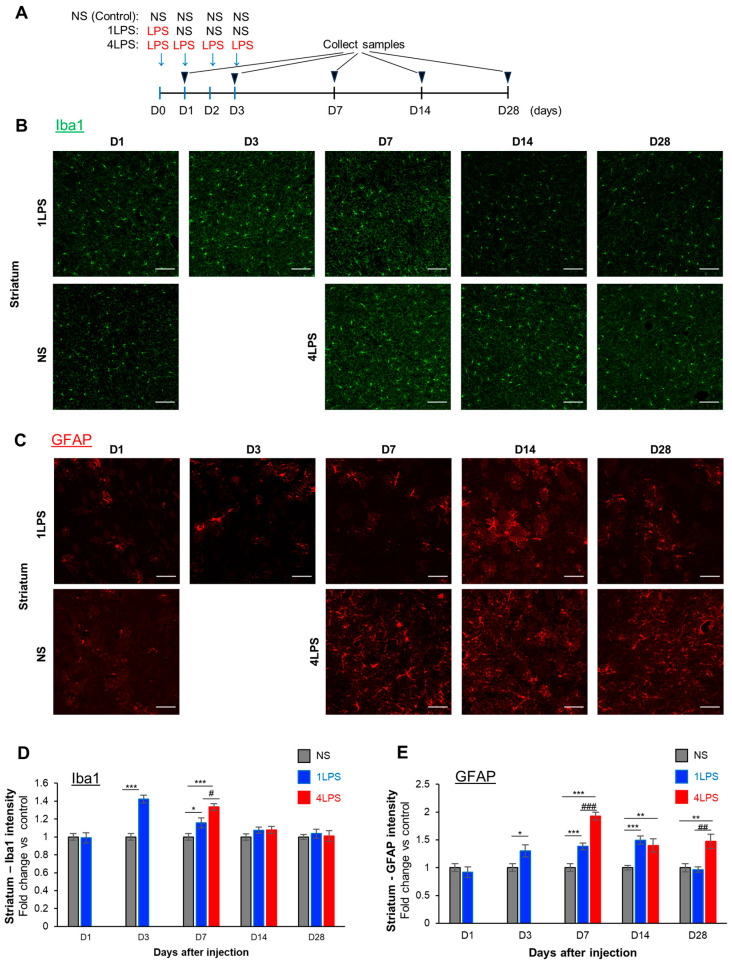
Time course of LPS administration-induced microglia and astrocyte activation in the striatum. (**A**) Experimental protocol for collecting IHC samples. Mice were injected with LPS for 4 consecutive days (4LPS group), for 1 day followed by 3 injections of normal saline (1LPS group), or 4 times with normal saline (control normal saline (NS) group). Brain samples were collected at D1 and D3 only for the 1LPS group, and D7, D14, and D28 for all groups. Representative images of Iba1 (**B**) and GFAP (**C**) staining in the striatum of all groups at different time points. Samples from NS group mice were collected at different time points but only one image is shown here. Scale bar = 100 µm. (**D**) Quantitative data for Iba1 intensity in the striatum of (**B**). Values are shown as the means ± SEM; * *p* < 0.05, *** *p* < 0.001 vs. NS; # *p* < 0.05 vs. 1LPS, *n* = 4 mice per group, one-way ANOVA with Tukey’s post hoc test; gray bar: NS, blue bar: 1LPS, red bar: 4LPS. (**E**) Quantitative data for GFAP intensity in the striatum of (**C**). Scale bars, 100 µm. Values are shown as the mean ± SEM; * *p* < 0.05, ** *p* < 0.01, *** *p* < 0.001 vs. NS, ## *p* < 0.01, ### *p* < 0.001 vs. 1LPS, *n* = 4 mice per each group, one-way ANOVA with Tukey’s post hoc test; gray bar: NS, blue bar: 1LPS, red bar: 4LPS.

We next examined how peripheral inflammation modulated cerebral infarction, and what type of cells mediated this action. Previous studies reported that LPS challenge activated glial cells, which affected various disease conditions [11]. We therefore considered that glial cells were involved in the LPS-induced exacerbation or protection of MCAO. MCAO-induced lesions were present in the striatum and cortex, and the effect of LPS was also observed in these brain regions, although its effect was particularly pronounced in the striatum (Figure 1). Therefore, we presented data specifically related to the striatum. We investigated the effect of LPS on the glial cells, microglia, and astrocytes. We harvested brain samples at 1, 3, 7, 14, and 28 days (D1, D3, D7, D14, and D28) after the first day of NS, 1LPS, or 4LPS i.p. injection and performed immunohistochemistry for Iba1 and GFAP (Figure 2A). In the striatum, 1LPS increased Iba1 intensity, which showed a rapid onset and transient response as it was increased significantly at D3 and D7 but declined to levels similar to the NS-treated controls at D14 and D28 (Figure 2B,D). The 4LPS also significantly increased Iba1 intensity at D7, which was transient and decreased to baseline levels after D14. At D7, Iba1 intensity in 4LPS mice was significantly higher than that in 1LPS mice. Similar results were obtained in the cerebral cortex, a brain region that is supplied by the MCA. Similar to the Iba1 intensity, LPS increased the intensity of GFAP staining in the striatum. GFAP intensity was significantly increased in the 1LPS group at D3, D7, and D14 and the 4LPS group at D7, D14, and D28 (Figure 2C,E). The onset of activation was similar to that of Iba1, but its duration was longer for GFAP, with increases observed up to D14 for 1LPS and up to D28 for 4LPS (Figure 2E). Similar results were obtained in the cerebral cortex. Collectively, the peripheral administration of LPS activated microglia and astrocytes, and the activation of microglia was more transient, consistent with previous studies [2].

### 2.3. Severe Inflammation by LPS Protects against MCAO via Microglia

**Figure 3:**
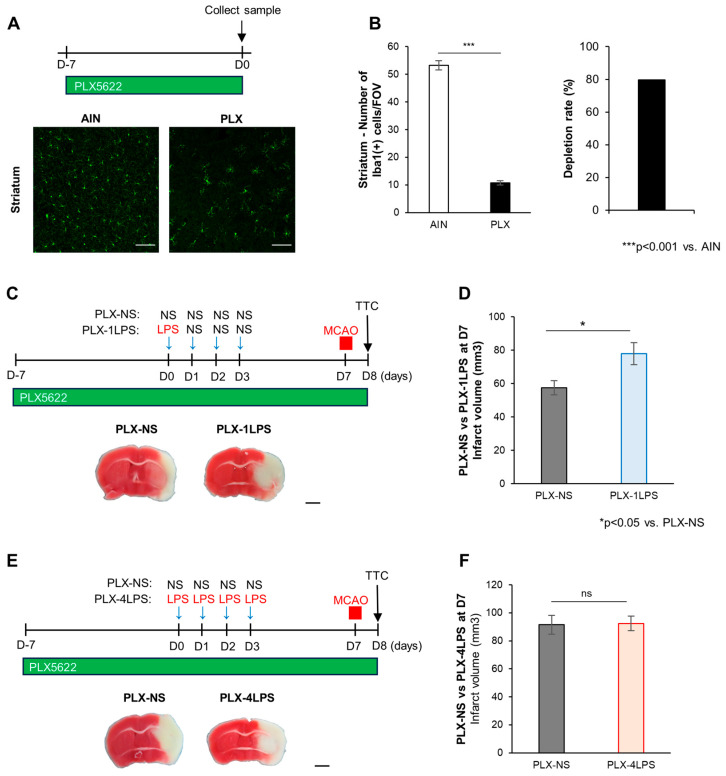
Effects of PLX treatment on 1LPS-induced exacerbation and 4LPS-induced protection of MCAO brain damage. (**A**) Top: Schematic diagram showing the experimental protocol for PLX efficacy. Bottom: Representative images of Iba1 staining in the striatum after 7 days of PLX diet and control diet (AIN). Scale bars = 100 µm. (**B**) Quantification of Iba1(+) cell numbers in one field of view (left) and the depletion rate (right) in the striatum, *n* = 3, *** *p* < 0.001, unpaired *t*-test. (**C**) Top: Schematic diagram showing the experimental protocol. Bottom: Representative images of TTC staining (PLX-normal saline (NS) group (left); PLX-1LPS group (right)). Scale bar, 2 mm. (**D**) Quantification of infarct volumes in PLX-NS mice (*n* = 6) and PLX-1LPS mice (*n* = 10) (PLX-NS mice vs. PLX-1LPS mice, * *p* < 0.05, unpaired *t*-test). (**E**) Top: Schematic diagram showing the experimental protocol. Bottom: Representative images of TTC staining (PLX-NS group (left); PLX-4LPS group (right)). Scale bar, 2 mm. (**F**) Quantification of infarct volumes in PLX-NS mice (*n* = 6) and PLX-4LPS mice (*n* = 8) (PLX-NS mice vs. PLX-4LPS mice, *p* = 0.91, unpaired *t*-test). All data are expressed as the mean ± SEM. n.s.: not significant.

The effects of 1LPS and 4LPS on MCAO were observed for a limited time window around D7, and this time course was similar to that of microglial activation. As microglial activation was observed at D7 after the 1LPS and 4LPS treatments, we investigated whether microglia were involved in the MCAO aggravating effect by 1LPS and the MCAO protective effect by 4LPS. For this, we depleted microglia using PLX5622 (PLX), an inhibitor of the colony-stimulating factor-1 (CSF1) receptor [12]. PLX was fed to mice from one week prior to and until the end of the experiment (Figure 3C,E). One week after starting PLX treatment, we conducted the same experiment as shown in Figure 1, and established 1LPS, 4LPS, and NS mice that subsequently underwent MCAO (Figure 3C,E). First, we confirmed that PLX feeding for 7 days prior to LPS administration reduced microglia in the mice brain by 80% (53.22 ± 1.68 and 10.78 ± 0.72 Iba1(+) cells in one field of view of the striatum for control diets and PLX diets, respectively, *p* < 0.001, Figure 3A,B). Next, we tested whether the depletion of microglia by PLX affected MCAO-evoked brain damage in NS-treated mice and confirmed that there was no significant difference in the MCAO-evoked infarct volume between NS and PLX-NS mice (PLX-NS: 82.8 ± 8.64% of the NS group, *p* = 0.17 with 30 min MCAO and PLX-NS: 102.4 ± 10.73% of the NS group, *p* = 0.675 with 40 min MCAO). Then, we investigated the effect of PLX on MCAO-evoked brain damage in 1LPS- and 4LPS-treated mice. As shown in Figure 3D, the MCAO exacerbating effect in 1LPS mice was not affected by PLX treatment. The MCAO-evoked infarct volume in 1LPS-treated mice was still significantly larger than that in NS mice, even under PLX treatment conditions (Figure 3D; PLX-NS vs. PLX-1LPS, *p* < 0.05). In contrast, the MCAO protecting effect of 4LPS was almost abolished by PLX treatment, and there was no significant difference in the infarct volume between PLX-NS and PLX-4LPS mice (Figure 3F; as in the Figure 1 data, control PLX-NS group for PLX-4LPS is substantially different from that for PLX-1LPS group because the MCAO duration time of two experiments is not identical; see Section 4 Materials and Methods). These data indicate that 1LPS-induced MCAO exacerbation was independent of microglia, whereas the 4LPS-induced MCAO protection was dependent on microglia, suggesting that different degrees of peripheral infection/inflammation have opposite effects on MCAO-evoked brain damage. Interestingly, the difference appeared to be related to the involvement of microglia.

### 2.4. Peripheral Macrophages Contribute to Mild Inflammation-Related MCAO Exacerbation

**Figure 4:**
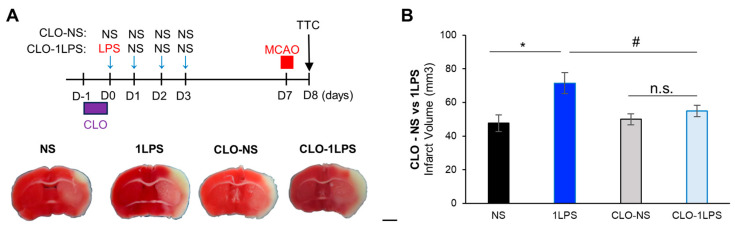
Clodronate (CLO) treatment abolishes 1LPS-induced exacerbation of MCAO lesions. (**A**) Top: Schematic diagram showing the experimental protocol. Bottom: Representative images of TTC staining in the non-CLO-normal saline (NS) group (*n* = 5 mice), non-CLO-1LPS (1LPS) group (*n* = 6 mice), CLO-NS group (*n* = 6 mice), and CLO-1LPS group (*n* = 8 mice). Scale bar, 2 mm. (**B**) Quantification of infarct volumes (NS mice vs. 1LPS mice, * *p* < 0.05; NS mice vs. CLO-NS mice, *p* = 0.70; 1LPS mice vs. CLO-1LPS mice, # *p* < 0.05; CLO-NS vs. CLO-1LPS, *p* = 0.41. One-way ANOVA with Tukey’s post hoc test). All data are expressed as the mean ± SEM. n.s.: not significant.

Our prior data suggested that MCAO exacerbation was independent of microglia. LPS administration activates peripheral immune cells that produce and release many active molecules such as cytokines and chemokines, leading to systemic inflammation. This LPS-induced inflammation also causes a proinflammatory state in the brain via released cytokines [2,13,14]. Therefore, to examine the mechanism underlying the 1LPS exacerbation, we targeted macrophages, a type of immune cell present in systemic organs or in the border of the brain, that are activated by the systemic injection of LPS [15], which might have an impact on brain parenchymal tissue through the release of cytokines that cross the blood-brain barrier. We used clodronate to deplete macrophages to determine whether they were required for MCAO exacerbation [16] in 1LPS mice (Figure 4A). Interestingly, clodronate-treated 1LPS mice did not show increased infarct volumes compared with non-clodronate-treated 1LPS mice (Figure 4B). In these mice, the infarct area was localized within the cortex of the MCAO ipsilateral side, and the striatum regions were spared (Figure 4A,B), suggesting that clodronate suppressed the 1LPS-induced MCAO exacerbation. Clodronate itself did not have any effects on the infarct size in NS control mice (Figure 4B). Therefore, this exacerbation requires macrophages outside or at the border of the brain.

### 2.5. Transcriptome Analysis of Microglia Identifies Pathways Related to LPS-Induced MCAO Protection

**Figure 5:**
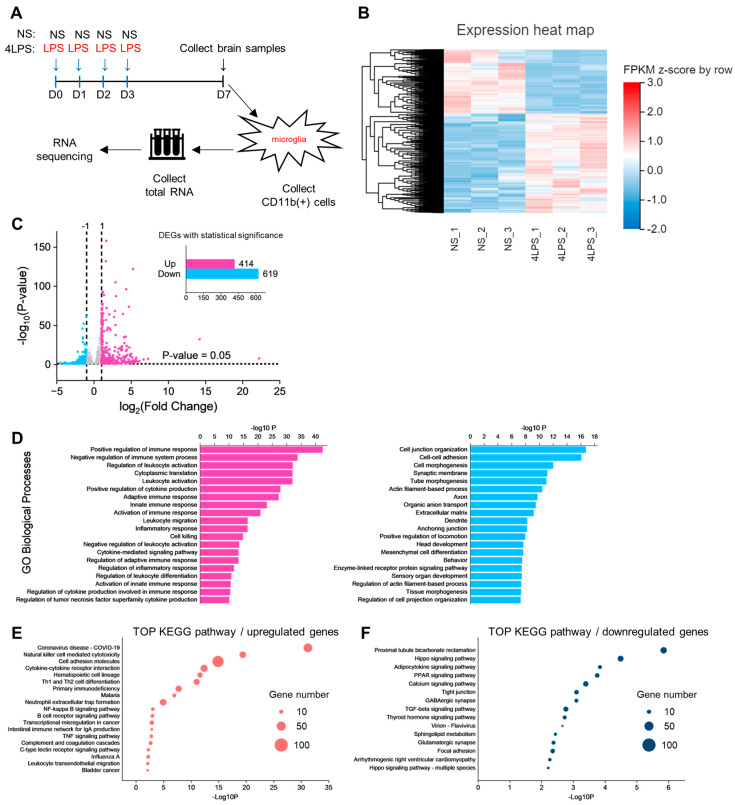
Transcriptome analysis of microglia isolated from 4LPS mice. (**A**) Schematic diagram illustrating the isolation of CD11b-positive cells from the cortex for RNA-seq using the MACS procedure. (**B**) Heatmap of z-scores obtained from the fragments per kilobase of exon per million mapped fragments (FPKM) of 2334 genes in the 4LPS group (*n* = 3 samples) and NS group (*n* = 3 samples). (**C**) Volcano plot of differentially expressed genes (DEGs) between microglia of the 4LPS group and NS control group (*x*-axis: log2 fold change, *y*-axis: −log10 adjusted *p*-value). Analysis of DEGs (4LPS vs. NS control) in microglia (upregulation of 414 genes and downregulation of 619 genes). (**D**) Top Gene Ontology (GO) terms of upregulated genes (**left**) and downregulated genes (**right**). (**E**,**F**) Top KEGG pathways of (**E**) upregulated genes and (**F**) downregulated genes. The dot size represents the number of genes with statistically significance. The horizontal axis is the *p*-value of the pathways analyzed.

To gain molecular insights into severe inflammation-induced (4LPS) MCAO protection, we performed the RNA sequencing (RNA-seq) analysis of microglia obtained from 4LPS-treated mice or NS-treated mice. We isolated CD11b^+^ cells from these mice at D7 using a magnetic-activated cell sorting (MACS) procedure (Figure 5A). As shown in a heatmap diagram, an overview of this transcriptome analysis revealed a global transcriptional change between NS mice and 4LPS mice (Figure 5B). We then performed an analysis of differentially expressed genes (DEGs) between the two groups. DEG analysis of microglia from 4LPS mice showed 414 upregulated genes and 619 downregulated genes (cutoff: |log2 fold change| > 1 with a *p*-value < 0.05) (Figure 5C). In 4LPS-treated microglia (vs. NS microglia), highly upregulated genes included *Saa3*, *Cd96*, and *Cxcl13*, relevant to LPS-induced inflammation [17,18]. Of note, expression of the *Cxcl13* gene was enriched in neurogenesis-related microglia and repopulated microglia [19,20,21]. However, the expression levels of classical proinflammatory genes (*Il1b*, *Tnf*, *Il6*, and *Ccl2*) were not increased whereas the expressions of some anti-inflammatory genes (*Il10*, *Dusp1*, and *Zfp36* [22,23,24,25]) were significantly upregulated, and other anti-inflammatory genes (*Socs3*, *Mrc1*, and *Arg1*) tended to be upregulated but without reaching statistical significance. Additionally, 4LPS-treated microglia had a high expression of *Itgax* (integrin αX/CD11c), a signature gene of disease-associated microglia (DAM) [26]. Furthermore, homeostatic genes (*Tmem119*, *Cx3cr1*, *Siglech*, *Csf1r*, *P2ry12*, *Sall1*, *Hexb*, and *Gpr34*) were not downregulated. In agreement with previous studies, these data indicate that 4LPS-treated microglia showed a transcriptional profile of anti-inflammatory and neuroprotection [13,14,19,27], which is distinct from typically acute LPS inflammation [1,2,28]. To understand genes with significantly altered expressions, we performed Gene ontology (GO) analysis. The GO analysis of upregulated genes showed that most GOs were involved in immune and inflammatory functions (Figure 5D). Similar to the GO analysis, KEGG pathway analysis demonstrated that 4LPS-treated microglia were enriched for several immune response-related pathways including “Cytokine-cytokine receptor interaction”, “Natural killer cell mediated cytotoxicity”, “NF-kappa B signaling pathway”, “Th1 and Th2 differentiation”, and “B cell receptor signaling pathway” (Figure 5E). Taken together, 4LPS-treated microglia exhibited a transcriptional shift related to neuroprotective anti-inflammation that may be associated with MCAO protection.

## 3. Discussion

Our findings in this study were as follows. (1) Pre-conditioning of mild peripheral inflammation (1LPS) exacerbated subsequent MCAO injury, which was independent of microglia although 1LPS activated microglia. (2) Pre-conditioning with severe peripheral infection (4LPS) reduced subsequent MCAO injury, indicating the so-called ischemia tolerance phenomenon, which was dependent on microglia. (3) The expressions of genes including *Cxcl13*, *Dusp1*, *Itgax*, and *Il10* were upregulated in microglia after 4LPS pre-conditioning, suggesting they were associated with microglia-dependent ischemic tolerance. Therefore, peripheral infection/inflammation affects brain function but regulates MCAO injury in opposite directions depending on the degree of infection/inflammation, and the presence or absence of microglial involvement has a major role in this difference.

### 3.1. Pre-Conditioning of Acute Mild Peripheral Inflammation (1LPS) Causes the Microglia-Independent Exacerbation of MCAO Injury

Acute mild peripheral inflammation in the 1LPS group caused hyperactive microglial responses and MCAO exacerbation. This exacerbation was unaffected by microglial depletion but the ablation of macrophages by clodronate abolished it, indicating the effect was independent of microglia but dependent on peripheral immune cells. However, since the first report [12], CSF1R inhibitors, such as PLX, have been used as a method of microglial depletion under the assumption that peripheral immune cells are not affected, it has been reported that PLX also affects some peripheral macrophages [29]. Thus, more precisely, the MCAO aggravating effect by 1LPS is microglia- and sometimes macrophage-independent, and clodronate-sensitive macrophage-dependent. As LPS cannot enter the BBB [13], it did not have a direct effect on microglia. LPS can directly activate macrophages and monocytes via Toll-like receptor 4 and CD14 to produce proinflammatory cytokines. These cytokines include tumor necrosis factor-α (TNF-α), IL-1, IL-6, and other biological mediators [15] that can cross the BBB to reach the brain [30,31] where they activate non-microglial cells such as astrocytes. Indeed, after 1LPS treatment, astrocytes become activated with an elevated expression of GFAP. Such astrocytes may further amplify the inflammation, leading to the subsequent exacerbation of MCAO, because some astrocyte phenotypes are highly neurotoxic [32]. In addition, peripheral immune cell-derived molecules might act on neurons and/or the brain vasculature, leading to more severe MCAO damage. However, further studies are needed to clarify the molecular mechanisms of the 1LPS-induced exacerbation of MCAO damage including which cells and macrophage-specific molecules are involved.

Although it was reported that clodronate selectively inhibited macrophages and microglia, it was recently reported that clodronate liposomes also affected neutrophils [33], which are the first cells to enter the brain following an ischemic event. Neutrophils, attracted to ischemic regions by cytokines, chemokines, and DAMPs released from the brain, cause secondary injury by releasing other inflammatory factors. Thus, we cannot exclude the possibility that the reduction in MCAO injury might be related to the reduction in neutrophils. Irrespective of this, clodronate-sensitive peripheral immune cells might exacerbate the damage caused by MCAO by the pre-conditioning of 1LPS.

### 3.2. Pre-Conditioning of Severe Peripheral Inflammation (4LPS) Causes Microglia-Dependent Inhibition of MCAO Injury

Cerebral ischemic tolerance is a clinically and experimentally observed phenomenon in which the brain, the most vulnerable organ to ischemia, acquires resistance to subsequent invasive ischemia after prior noninvasive ischemia (pre-conditioning). This phenomenon was first discovered by Murry et al. in 1986 in a study of the heart [34]. The cardioprotective effect of ischemic tolerance is very strong and it has been observed in many organs other than the heart, including the lungs, kidneys, liver, skeletal muscle, and brain [35,36,37]. Therefore, many studies have attempted to elucidate the molecular mechanisms of ischemic tolerance as a key to therapeutic strategies for stroke. Although several important molecules and intracellular signals have been reported to induce cerebral ischemic tolerance [38,39,40,41], most involve neurons. It is important that the pre-conditioning stimulus to induce ischemic tolerance does not necessarily have to be an ischemic load. Instead, physical stimuli such as electronic stimulation, chemicals such as 3-nitropropionic acid [42] and resveratrol [43], and infectious stimuli such as LPS [27,44,45,46,47,48] have been used. These types of ischemic tolerance are termed cross-ischemic tolerance [37]. For medical applications, “prior mild ischemic loading” is not realistic. Therefore, the discovery of cross-ischemic tolerance is important for research with a view to clinical applications, and at the same time, it is very useful for elucidating the molecular mechanism of ischemic tolerance, which is still largely unknown.

In this study, pre-conditioning of 4LPS induced brain ischemic tolerance against MCAO, which, unlike 1LPS, was dependent on microglia because this inhibitory action against MCAO was abolished when microglia were depleted by PLX (Figure 3E,F). We previously showed that pre-conditioning of mild non-invasive MCAO caused ischemic tolerance against the subsequent lethal MCAO, which was independent of microglia but dependent on astrocytes [49,50,51]. The mechanism of this astrocyte-dependent cerebral ischemic tolerance was that pre-conditioning with mild MCAO increased P2X7R expression on astrocytes, which produced various neuroprotective factors via HIF1α. Microglia were required for the induction of ischemic tolerance by astrocytes, but microglia per se were not necessary for ischemic tolerance. Therefore, the results of this study using LPS for pre-conditioning seem to contradict previous studies on ischemic tolerance using mild MCAO as pre-conditioning. The difference in the type of pre-conditioning, i.e., brief MCAO and 4LPS, may have contributed to this difference, but the molecular mechanisms involved remain unclear. Presumably, a molecular and cellular signal was activated by 4LPS to promote microglia toward a neuroprotective phenotype, but further studies are needed to elucidate the detailed mechanism. Additionally, recent studies have shown that the recruitment of monocytes that occurs in post-MCAO lesions can be relevant to the alleviation of infarct damage [52,53]. It cannot be excluded that post-MCAO-monocyte infiltration may be partially involved in MCAO protection in our study. Furthermore, 1LPS had a microglia-independent MCAO exacerbation effect, whereas 4LPS had a microglia-dependent MCAO suppressive effect. Although the reasons for this difference are unknown, the intensity of peripheral infection and/or inflammation has opposite effects on subsequent MCAO damage, which might be attributable to the involvement of microglia. This will be important when considering the linkage between peripheral inflammation and brain functions in the future.

### 3.3. Molecular Mechanisms Underlying 4LPS-Induced Microglia-Dependent Ischemic Tolerance against MCAO

Several studies have investigated microglia-mediated neuroprotective pre-conditioning. A preceding LPS stimulus reduced ischemic infarct lesions [13,45,46,47,48]. After LPS initiates inflammatory responses, the innate immune system becomes hypoactive to subsequent ischemic events. This may be related to anti-inflammatory cytokines produced by the primary LPS challenge [44]. Previous studies suggest the involvement of *Socs3*, *Msr1*, *Cd163*, *Mrc1*, *Arg1*, *Il10*, and *Cxcl13* in LPS-induced pre-conditioning [19,27,54]. Our RNA-seq analysis showed similar data to these studies. Interleukin (IL)-10 injection reduced the brain infarct size caused by MCAO [55]. CXCL13, a neurogenesis- and tissue repair-associated molecule, was produced in microglia treated multiple times with LPS [19]. In particular, *Itgax* was upregulated in 4LPS-treated microglia, which has rarely been reported in previous studies of LPS pre-conditioned microglia. *Itgax*/CD11c-positive microglia were reported to be increased in neurological disease models [56], and *Itgax* was recently identified as a DAM gene [26,57]. CD11c-positive microglia are key players in myelination and neurogenesis in the developing brain [56]. Furthermore, they are essential for the repression of neuropathic pain [58], which could indicate their neuroprotective functions. Therefore, the upregulation of these molecules may contribute to 4LPS-induced MCAO protection. Under ischemic conditions, microglia sense the microenvironmental milieu and change their gene expression profiles and functions accordingly, to protect against ischemic injury. RNA-seq of microglia from mice with LPS/MCAO may enhance our understanding of the MCAO protection mechanism.

### 3.4. Limitations of the Study

Since LPS only simulates one type of bacterial infection, it is not known whether the same mechanism regulates brain function in various other types of infection. In addition, the degree of infection is not as simple as 1LPS and 4LPS, making it difficult to extrapolate the relationship between the experimental models and actual infection/inflammation. These are the limitations of this study. However, it is clear that the degree of peripheral inflammation affects brain function differently, depending on whether microglia are involved, which is important.

### 3.5. Perspective

The heterogeneity of the microglial population has been reported previously in terms of their morphology, functions, and transcriptions. We conducted bulk RNA-seq of LPS-treated microglia, which cannot detect diverse transcriptome changes in microglial subpopulations. Single-cell RNA sequencing of 4LPS-treated microglia may contribute to a better understanding of the LPS pre-conditioning mechanism. In addition, as we mentioned above, we did not perform RNA-seq of microglia from 4LPS-treated mice that underwent MCAO. MCAO stimulation induces microglial phenotypic and transcriptional changes in microglia, and then microglia respond to and participate in the ischemic lesion. Therefore, RNA-seq data from LPS-treated microglia with MCAO is also required to elucidate the molecular mechanism of LPS-induced MCAO protection. We should optimize an experimental condition to obtain high-quality samples from MCAO lesions for future research.

## 4. Materials and Methods

### 4.1. Mice

All animal care and experimental procedures were conducted in accordance with the “Guiding Principles in the Care and Use of Animals in the Field of Physiologic Sciences” published by the Physiologic Society of Japan and with prior approval of the Animal Care Committee of the University of Yamanashi (Chuo, Japan). All experiments in this study used 8–10-week-old wild-type (WT) male mice (C57BL/6J) purchased from Japan SLC (Hamamatsu, Japan). All mice were maintained in a pathogen-free, temperature-controlled (23 °C) and humidity-controlled (55%) facility with a 12-h light/dark cycle. Mice had free access to food and water.

### 4.2. Lipopolysaccharide (LPS) Administration

LPS (*Escherichia coli* 055:B5, Sigma-Aldrich, St. Louis, MO, USA) was dissolved in saline at a concentration of 500 µg/mL, divided into small aliquots, and stored at −30 °C until use. LPS was defrosted and diluted to a 50 µg/mL solution using normal saline. Mice were intraperitoneally (i.p.) injected with LPS at a dose of 500 µg/kg body weight [13]. For the control, the same amount of normal saline (0.1 mL for each 10 g body weight) was injected into the peritoneum. Mice that received an LPS injection once daily for four consecutive days were used as a model of severe inflammation (4LPS group) [13,27]. Mice received a single LPS i.p. injection followed by normal saline i.p. injections once daily for the next three consecutive days as a model of acute mild inflammation (1LPS group). Control mice were injected with normal saline for four consecutive days (NS group). A much higher dose of LPS (more than 1 mg/kg) may cause a behavior alteration, septic shock, or a decrease in food and water intake, associated with sickness behavior or depressive-like behavior. These physical and behavioral changes often lead to systemic deterioration, body weight loss, or dehydration symptoms that can influence brain infarct lesions. To avoid these complications, we selected this dose of LPS.

### 4.3. Middle Cerebral Artery Occlusion (MCAO)

Unilateral transient focal ischemia in mice was induced by intraluminal filament occlusion of the right middle cerebral artery, as previously described [49]. The mice were anesthetized with 4% (*v*/*v*) isoflurane and maintained on 1.25% (*v*/*v*) isoflurane with a facemask. Mice were placed in a supine position and the four limbs were fixed with medical tape. After making an incision in the neck, the common carotid artery (CCA), internal carotid artery (ICA), and external carotid artery (ECA) were exposed by dissection. The ECA and distal and proximal position of the CCA were ligated, and a 15 mm length of 6-0 silicone rubber-coated monofilament suture (602356PK10Re, Doccol, Sharon, MA, USA) was inserted via the CCA into the ICA and then into the circle of Willis, thereby occluding the MCA. The MCA was occluded for 30 min in 1LPS experiments and for 40 min in 4LPS experiments, then the suture was carefully withdrawn to allow reperfusion of the ischemic region. Mice were allowed to recover in a warm environment for 1 h and sacrificed 24 h after MCAO for the histological examinations.

### 4.4. Drug Treatment

PLX5622 (PLX; 1200 ppm) was obtained from Amadis Chemical (Hangzhou, China), and formulated in AIN-76A standard chow. Mice were fed PLX 7 days before injection to the end of the experiment. Clodronate liposome (anionic) (Clophosome F70101C-A, Funakoshi, Tokyo, Japan) was stored at 4 °C, warmed to room temperature, and mixed well before use. Mice were i.p. injected with 200 µL clodronate 1 day before normal saline or LPS injection [33,59].

### 4.5. Immunohistochemistry

Anesthetized mice were intra-cardially perfused with ice-cold phosphate-buffered saline (PBS), followed by perfusion with ice-cold 4% PFA. Brains were extracted and post-fixed in 4% PFA overnight at 4 °C, cryoprotected with 30% sucrose in PBS, and embedded in an OCT compound (Sakura Finetek, Tokyo, Japan). Frozen sections were cut at 20 µm thickness on a cryostat (CM1520, Leica, Wetzlar, Germany) and stored in cryoprotectant at −30 °C. For staining, floating sections were washed three times with PBS and blocked with 10% normal goat serum in PBS containing 0.5% Triton X-100 at room temperature for 1 h. The slices were then incubated with primary antibodies in PBS for 48 h at 4 °C. The primary antibodies used were as follows: rat anti-GFAP (1:500; Invitrogen, Waltham, MA, USA) and rabbit anti-Iba1 (1:1000; Wako, Osaka, Japan). Slices were washed three times with PBS and incubated with Alexa fluor 488-conjugated goat anti-rabbit IgG (1:1000; Life Technologies, Waltham, MA, USA) or Alexa fluor 546-conjugated goat anti-rat IgG (1:1000; Life Technologies) for 2 h at room temperature. Images were obtained by a confocal microscope (FluoView1200; Olympus, Tokyo, Japan) equipped with 20× and 40× objective lenses (N.A. 0.75).

### 4.6. TTC Staining for Measure of Cerebral Infarct Size

Mice were anesthetized and intra-cardially perfused with saline. The brains were removed, and the forebrain was sliced into 1 mm thick coronal sections. The sections were stained with 2% (*w*/*v*) 2,3,5-triphenyltetrazolium chloride (TTC; Sigma-Aldrich) saline solution at 37 °C for 10 min. After staining, images of slices were captured by a digital camera (SX 710HS, Canon, Tokyo, Japan). The infarct size was calculated as the summation of the infarct volume of all slices.

### 4.7. Magnetic Cell Separation

We used a magnetic cell sorting method to isolate microglia from brain tissues. All procedures are based on the MACS^®^ procedure of Miltenyi Biotec (Bergisch Gladbach, Germany), as previously described [60]. Briefly, the cortices were removed seven days after the first day of LPS or normal saline daily i.p. injection for four consecutive days. Dissected brain tissue was dissociated using a MACS^®^ Adult Brain Tissue Dissociation Kit (Miltenyi Biotec, Bergisch Gladbach, Germany). Following the removal of cell debris, dissociated cells were re-suspended in 0.5% BSA in PBS. Next, an anti-CD11b MicroBead Kit (Miltenyi Biotec) was used to isolate microglia from the cell suspension. After the cell suspension was centrifuged, the supernatant was completely aspirated and 0.5% BSA in PBS was added to the cell pellet. Fcr Blocking Reagent (Miltenyi Biotec) was then added to the cell suspension followed by anti-CD11b microbeads and the cell suspension was incubated for 15 min at 4 °C to achieve magnetic labeling. The cell suspension was then applied to the MACS^®^ column (Miltenyi Biotec), which was placed in the magnetic field of the MACS^®^ separator (Miltenyi Biotec). Unlabeled cells passed through and were collected. The magnetically-labeled cells were retained within the column. The column was removed from the separator and placed in a collection tube. An appropriate amount of buffer was added to the column and the magnetically labeled cells were eluted as the positively-selected cell fraction. After centrifugation (5000× *g* for 3 min) and removal of the supernatant, total RNA was purified from the cell pellets using an RNeasy^®^ Micro Kit (Qiagen, Venlo, The Netherlands). Each RNA sample was obtained from two mice. Three RNA samples per group were prepared.

### 4.8. RNA Sequencing Analysis

The quality of RNA samples was assessed using an RNA 6000 Pico Kit (Agilent, Santa Clara, CA, USA) and a 2100 Bioanalyzer (Agilent). First, 1 µg of total RNA was used to construct sequencing libraries. The sequencing libraries for RNA-seq were prepared using the Hieff NGS™ Ultima Dual-mode mRNA Library Prep Kit (Yeasen Biotechnology, Shanghai, China) according to the manufacturer’s protocol and then sequenced with DNBSEQ-G400 (MGI Tech, Shenzhen, China) based on the PE150 strategy. The sequencing data was filtered with SOAPnuke (v1.5.2) to remove reads containing a sequencing adapter, those with low quality, and those with a greater than 5% unknown base (‘N’ base) ratio. Clean reads were obtained and stored in the FASTQ format. The subsequent analyses and data mining were performed on the Dr. Tom Multi-omics Data mining system (https://biosys.bgi.com/, accessed on 12 July 2023). The clean reads were mapped to the reference genome of *Mus musculus* (UCSC mm39) using HISAT2 (v2.0.4) and Bowtie2 (v2.2.5), respectively. Then, Ericscript (v0.5.5) and rMATS (v3.2.5) were used to detect fusion genes and differential splicing genes (DSGs), respectively. Expression levels of genes were calculated by RSEM (v1.2.8). The differential expression analysis was performed using DESeq2 (v1.16.1). Gene enrichment analysis (Gene Ontology term analysis and KEGG pathway analysis) was conducted using the Metascape website (https://metascape.org, accessed on 1 August 2023). Gene expressions with 1 more than |log2 fold change| and a *p*-value less than 0.05 were considered significantly changed.

### 4.9. Statistical Analysis

Statistical analysis was performed using the Student’s *t*-test for comparisons between two groups and one-way ANOVA followed by Tukey’s post-hoc test for comparisons among three or more groups. Differences were considered significant when the *p*-value was less than 0.05. Data are expressed as the means ± the standard error of the mean (SEM). OriginPro 2021 or 2022 (Origin-Lab, Northampton, MA, USA) was used for the statistical analyses.

## 5. Conclusions

We demonstrated that the degree of peripheral infection and/or inflammation had the exact opposite effect of exacerbating or alleviating cerebral ischemic injury. Mild inflammation by 1LPS exacerbated the cerebral ischemic injury, which was independent of microglia, but was dependent on peripheral immune cells. In contrast, severe inflammation by 4LPS alleviated cerebral ischemic injury, which was dependent on microglia. Thus, systemic inflammation differentially affects the brain through the coordination of peripheral immune and glial cells.

## Data Availability

The data that support the findings of this study are available from the corresponding author upon reasonable request. The sequencing data reported in this paper are available through the NCBI Gene Expression Omnibus (GEO) accession GSE244358.
